# Post-Activation Potentiation’s Impact on Specialized Strike Strength in Elite Male Boxers: An Acute Study

**DOI:** 10.3390/s25206356

**Published:** 2025-10-14

**Authors:** Yongfu Liu, Rangxi Jin, Chao Chen

**Affiliations:** 1 School of Physical Education, University of Jinan, Jinan 250022, China; 18721790956@163.com; 2 School of Athletic Performance, Shanghai University of Sport, Shanghai 200438, China; 2421852041@sus.edu.cn; 3 College of Physical Education, Dalian University, Dalian 116622, China

**Keywords:** straight punch striking strength, activation intensity, peak striking power, optimal activation point

## Abstract

**Objective:** The purpose of this study is to investigate the effects of back squat (BS) and squat jump (SJ) on the maximum-striking strength and speed-striking strength of the jab and cross of elite male boxers, and to identify the time point of the post-activation performance enhancement (PAPE) induced by these two activation methods. **Methods:** A total of 29 Chinese male boxers were recruited to participate in four different intensities of muscle activation through BS and SJ exercises (BS_50%_, SJ_50%_, BS_80%_, SJ_80%_). The participants were tested on their jab and cross using specialized testing protocols at recovery intervals of 4, 8, 12, and 16 min (speed-striking strength testing was conducted first, followed by maximum-striking strength testing), and the maximum-striking strength and speed-striking strength of the athletes were recorded. **Results:** (1) Maximum-striking strength: For the jab, the results indicated that there were significant differences between BS_50%_ at 8 min and 12 min and the baseline (*p* < 0.01), and between SJ_50%_ at 4, 8, and 12 min and the baseline (*p* < 0.01). BS_80%_ showed significant differences at 12 min compared to baseline (*p* < 0.01), and the SJ_80%_ exhibited significant differences at 8 min (*p* < 0.05) and 12 min (*p* < 0.01) compared to baseline. For the cross, BS_50%_ demonstrated significant differences at 12 min compared to baseline (*p* < 0.01), and SJ_50%_ showed significant differences at 8 min and 12 min (*p* < 0.01). Both BS_80%_ and SJ_80%_ revealed significant differences at 8, 12, and 16 min compared to baseline (*p* < 0.01). (2) Speed-striking strength: For the jab, there were no significant differences between BS_50%_ and SJ_50%_ at all time intervals compared to baseline (*p* > 0.05). BS_80%_ showed a significant difference at 4 min compared to baseline (*p* < 0.05), and SJ_80%_ exhibited significant differences at 12 min compared to baseline (*p* < 0.01). For the cross, there were no significant differences between BS_50%_, SJ_50%_, and BS_80%_ at all time intervals compared to baseline (*p* > 0.05), while SJ_80%_ demonstrated significant differences at 8 min and 12 min compared to between (*p* < 0.01). The results showed that PAPE significantly enhanced maximum punch force at 8–12 min across several activation conditions. In contrast, improvements in speed-striking force were only observed following high-load squat jump (SJ at 80% 1 RM), with significant increases at 8 min for the cross and at 12 min for the jab, whereas BS or lower-load SJ produced no meaningful changes. **Conclusions:** PAPE activation significantly enhances the striking force of boxers at the recovery interval of 12 min, but the effect is influenced by the intensity and method of activation. High-load activation can enhance the striking strength of boxers more rapidly and sustainably, and high-load SJ are more beneficial for the speed-striking strength of boxers.

## 1. Introduction

The primary objective of boxing is to successfully land a clean and crisp punch on the opponent without retaliation (i.e., to control the match, knock down, or defeat the opponent) [1]. Punching (such as a straight, swing, or hook) is a rapid, whole-body, multiplanar muscular movement that accelerates first towards the opponent’s head or torso [2]. Achieving a high level of striking force is crucial for boxers because it can increase the probability of winning by influencing the referee’s perception of a clear punch, weakening the opponent’s combat ability, and knocking down or defeating the opponent [2,3]. A study found that male boxers’ punches (straights and hooks) are executed through the coordinated effort of the entire body’s muscles, primarily initiated by the legs pushing off the ground, generating torque through the hips, torso, and upper body [4]. During the rotation, muscles are sequentially activated from the bottom up to maximize the speed and force of the attack [5]. Therefore, leg strength significantly impacts a boxer’s striking ability. Pre-competition training and warm-up activities play a crucial role in an athlete’s technical and tactical performance. In recent years, there has been a growing emphasis on utilizing post-activation performance enhancement (PAPE) [6] as a method to effectively stimulate an athlete’s neuromuscular system, promptly enhance specific abilities, and optimize the execution of tactics and techniques.

PAPE is a phenomenon where pre-stimulation induces a “contraction trace” in muscles, leading to a temporary and rapid increase in explosive power during subsequent movements. Ballistic Exercise (BE) and Heavy-Resistance Exercise (HRE) are common methods for inducing PAPE, with the Squat Jump (SJ) and Back Squat (BS) being the typical exercises for BE and HRE, respectively [7,8]. Studies have shown that BE can improve the specific physical performance of judo athletes [9], while HRE can enhance the peak punching power of kickboxing athletes and the technical performance of taekwondo athletes in spinning kicks [10]. It can be observed that both BE and HRE can induce PAPE in athletes of combat sports, and SJ training [7] and BS training [11] have also been confirmed to enhance the athletes’ lower limb maximum strength and explosive power. Previous research indicates that PAPE effects are more likely to occur when high loads (70–90% 1 RM) are used with recovery intervals of about 4–12 min, whereas low loads (<60% 1 RM) often fail to elicit meaningful improvements [12,13]. These findings support the rationale for including both BS and SJ at two intensities (50% and 80% 1 RM) and testing multiple recovery intervals (4–16 min) in the present study to evaluate their effects on boxing-specific performance. In addition to maximum punch force, this study also examined “speed-striking force,” defined as the ratio of peak force to the time required to reach it (Fmax/ΔT). Conceptually, this parameter reflects the neuromuscular system’s ability to generate high levels of force in the shortest possible time, similar to the rate of force development (RFD) widely discussed in the strength and conditioning literature [14,15]. In boxing, this capacity is particularly relevant because rapid force expression underpins the effectiveness of successive punches, combinations, and counterattacks. Previous studies have shown that higher punch force and faster force application can determine competitive outcomes in boxing matches [16]. Therefore, speed-striking force provides a meaningful indicator of both explosive capacity and combat effectiveness in this sport.

In boxing, the ability to generate explosive punching power at critical moments directly influences competitive outcomes [16,17]. Beyond training optimization, a key practical concern is whether post-activation performance enhancement (PAPE) can be harnessed as part of pre-competition preparation. Unlike laboratory-based warm-ups, boxers typically perform structured pre-bout warm-ups that are followed by an unavoidable waiting period, including organizational procedures, referee checks, and ring entrance, which usually results in a 5–15 min delay before the bout begins [8,13]. Such a time lag may coincide with the recovery window in which PAPE effects emerge, raising the possibility that well-structured conditioning activities could translate into enhanced punching force and velocity at the start of competition. Considering that a bout may last 3–12 rounds (approximately 9–36 min), even modest improvements in striking performance during the initial rounds may provide a meaningful tactical advantage. Therefore, clarifying the time course and magnitude of PAPE effects in boxing is of both scientific and practical relevance.

However, it is necessary to further investigate whether different activation methods and various load intensities can induce PAPE in boxers, determine if SJ is superior to BS, and explore potential differences in the timing of PAPE induction by the two activation methods. The purpose of this study is to explore the effects of BS and SJ on the maximum-striking force and rapid striking force of front and rear straight punches, and to identify the timing of peak PAPE induction by the two activation methods.

## 2. Materials and Methods

### 2.1. Experimental Approach to the Problem

A cross-sectional experimental study was conducted to compare the acute effects of different intensities and forms of resistance training activation on the maximum-striking force and rapid force in boxers. The entire experiment was completed within two weeks, with a total of four PAPE activations performed using squat jump at 50% 1 RM (SJ_50%_), squat jump at 80% 1 RM (SJ_80%_), back squat at 50% 1 RM (BS_50%_), and back squat at 80% 1 RM (BS_80%_). Each induction was spaced 48 to 72 h apart. All tests were conducted in a laboratory setting, under the direct supervision of researchers, with each participant performing under controlled environmental conditions at the same time of day (±1 h). This study was approved by the Shanghai University of Sport Scientific Research Ethics Committee for Human Experiments (approval number 102772021RT102).

### 2.2. Participant

Using G*Power (version 3.1.9.7, Heinrich-Heine-Universität Düsseldorf, Düsseldorf, Germany) to calculate the sample size, with “F test” selected for the test family and “ANOVA: Repeated measures, within factors” chosen as the statistical method, a confidence level of 95%, a two-tailed alpha of 0.05, a statistical power (β) of 0.95, one group, and 17 measurement occasions, it was calculated that a minimum of 15 samples are required [18].

A total of 29 Chinese male boxers (height, 178.08 ± 7.70 cm; weight, 69.69 ± 12.42 kg; age, 23.46 ± 2.17 years; training experience, 9.15 ± 1.71 years; back squat 1RM, 130.4 ± 27.72 kg) were recruited as participants. All athletes held the qualification of National Master Sportsman and competed in the middleweight category. All participants had not sustained any upper or lower limb injuries in the past month and were able to proficiently perform weighted half squat jumps and weighted back squats.

### 2.3. Experimental Design

**Figure 1:**
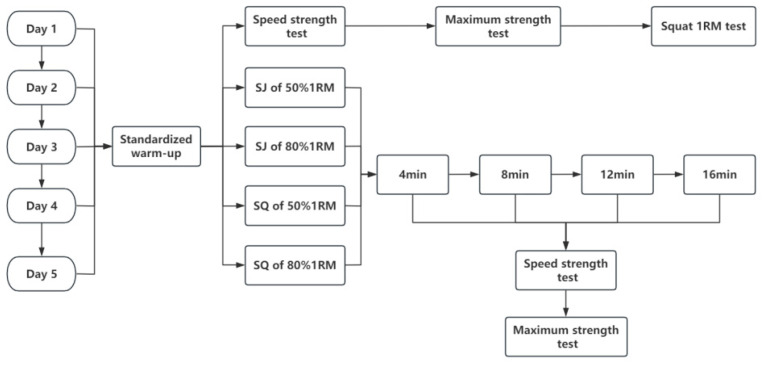
Test flow chart.

The study consisted of a total of five testing days, with intervals of 48–72 h between each testing day. Prior to testing, a standardized warm-up of 20 min was conducted, which included both general and specific warm-up exercises. The warm-up consisted of an initial 5 min jog, followed by a 10 min lower-limb muscle activation protocol comprising bodyweight squats (3 × 12 repetitions), walking lunges (2 × 20 m), lateral shuffles (2 × 15 m), vertical jumps (2 × 8 repetitions), and dynamic leg swings with joint mobility drills (~2 min), and concluded with 5 min of shadowboxing involving both front- and rear-hand straight punches. This protocol was confirmed by the boxing coaches to be consistent with the athletes’ regular training and pre-competition warm-up routines. On the first testing day, baseline assessments were conducted for the speed strike strength test, maximum strike strength test, and back squat 1 RM test. The remaining four testing days involved the activation induction with 3 sets × 5 times (with a 2 min rest between sets) of SJ_50%_, SJ_80%_, BS_50%_, and BS_80%_, respectively. For the activation protocol, participants performed 3 sets of 5 repetitions with a 2 min inter-set rest. To ensure consistency of the activation stimulus, strict exercise standards were applied. For the back squat (BS), athletes descended until the hip joint reached approximately 90° of knee flexion (thighs parallel to the ground) and then returned to full extension, with the barbell placed across the shoulders. For the squat jump (SJ), participants started from the same 90° knee flexion depth, paused for ~1 s to eliminate the stretch-shortening cycle, and then performed a maximal vertical jump while keeping their hands on the hips to avoid arm contribution. The jumps were required to be performed with maximal effort, with verbal encouragement provided to ensure maximal jump height. Following the activation, participants wore StrikeTec Boxing Sensors (StrikeTec, Dallas, TX, USA; version 1.4.4) along with boxing gloves. The StrikeTec boxing sensor (StrikeTec, USA) was used to measure punch force parameters. To ensure measurement accuracy, the device was calibrated before each testing session according to the manufacturer’s guidelines. Specifically, the sensor was reset to zero and tested against standard loads (5 kg and 10 kg) dropped vertically to verify output consistency. This calibration was performed prior to each day of testing. Participants weighing less than 69 kg wore 10-oz gloves, while those weighing 69 kg or more wore 12-oz gloves. They underwent specific tests for front and rear hand straight punches at recovery intervals of 4, 8, 12, and 16 min. Rapid force tests were conducted first, followed by maximum force tests (Figure 1).

### 2.4. Outcome Measures

#### 2.4.1. Back Squat 1 RM Test

Following a standardized warm-up session, participants initially completed 6 to 10 repetitions at an approximate load of 50% 1 RM, with a 2 to 3 min rest. Subsequently, they executed 3 repetitions at a heavier load, roughly equivalent to 80% 1 RM, followed by a 3 min recovery period. This process of incrementally increasing the load continued until the participant achieved their 1RM. Upon a successful lift, the weight was further increased by 5 to 10% of their 1 RM. In the event of a failed attempt, the load was adjusted downward by 5% 1 RM, with an accompanying 5 min rest. Each participant was permitted up to 5 attempts to determine their back squat 1 RM, with continuous and robust verbal encouragement provided throughout the testing procedure [19].

#### 2.4.2. Speed Strike Strength and Maximum Strike Strength Test

Participants adopted a standard boxing stance as they stood in front of a punching bag. Upon the tester’s command of “start”, the StrikeTec-Boxing Performance Tracking System was initiated. Concurrently, participants performed a test involving front-hand straight punches followed by rear-hand straight punches, each executed three times according to standard punching techniques. During the rapid force test, participants were instructed to strike as quickly as possible. The system displayed the force and time for each punch, which were then translated into a measure of rapid force (F_max_/ΔT). The best result was recorded with force values measured to the nearest 1 kg and time to the nearest 0.001 s. For the maximum force test, participants were asked to exert their full strength with each punch. The highest values for both front and rear straight punches (F_max_) were selected for recording, with force values measured to the nearest 1 kg.

### 2.5. Statistical Analysis

All data were analyzed statistically using SPSS 26.0, with results presented as mean values and standard error of the mean (SEM). The Shapiro–Wilk test was employed to assess the normality of all variables, Levene’s test was used to examine homogeneity of variances, and Mauchly’s test was conducted to assess sphericity. Repeated measures analysis of variance (ANOVA) was utilized to statistically analyze the pre, 4 min, 8 min, 12 min, and 16 min time points for both BS and SJ conditions. Depending on the outcomes of Levene’s test for homogeneity of variances and Mauchly’s test for sphericity, if *p* > 0.05, the univariate analysis results were adopted; otherwise, the Greenhouse–Geisser correction was applied to the univariate analysis results. A *p*-value of less than 0.05 was considered to indicate statistical significance in the univariate analysis results.

The reliability analysis demonstrated good test–retest consistency for both jab and cross punching force measurements. Specifically, the ICC (3.1) values ranged from 0.778 to 0.837 across conditions, indicating acceptable to good reliability. The SEM ranged from 9.44 to 17.46 N, while the CV% values varied between 9.13% and 12.52%. These findings confirm that the StrikeTec sensor provided stable and consistent assessments of jab and cross punching force.

## 3. Results

### 3.1. Jab

The results from the 50% 1 RM activation demonstrated significant differences at 8 min and 12 min compared to the baseline after BS activation (*p* < 0.01), as well as at 4 min, 8 min, and 12 min after SJ (*p* < 0.01). Adjacent time point comparisons revealed significant differences following BS activation between 4 min and 8 min (4 min < 8 min, *p* < 0.01) and between 12 min and 16 min (12 min > 16 min, *p* < 0.05). Similarly, significant differences were observed following SJ activation between 4 min and 8 min (4 min < 8 min) and between 12 min and 16 min (12 min > 16 min, *p* < 0.01). Comparisons at the same time points indicated no significant differences between BS and SJ activations (*p* > 0.05).

**Figure 2:**
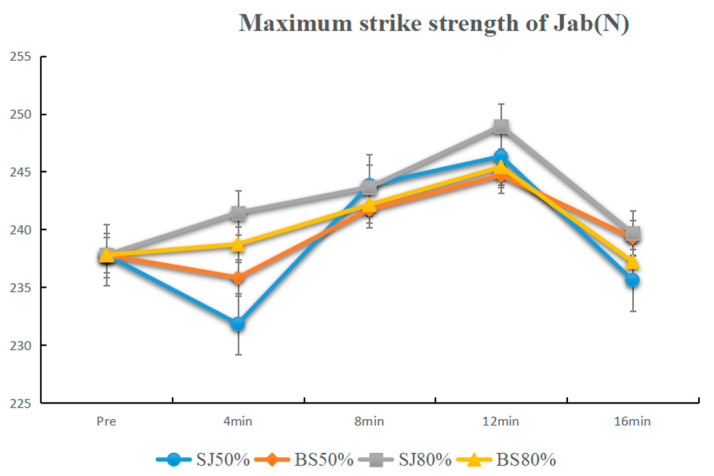
Line plot of the maximum strike strength of jab.

The results from the 80% 1 RM activation demonstrated significant differences at 12 min compared to the baseline after BS activation (*p* < 0.01), and at 8 min (*p* < 0.05) and 12 min (*p* < 0.01) after SJ (*p* < 0.01). Adjacent time point comparisons revealed significant differences following BS activation between 4 min and 8 min (4 min < 8 min, *p* < 0.05) and between 12 min and 16 min (12 min > 16 min, *p* < 0.01). Similarly, significant differences were observed following SJ activation between 8 min and 12 min (8 min < 12 min, *p* < 0.05) and between 12 min and 16 min (12 min > 16 min, *p* < 0.01). However, comparisons at the same time points showed no significant differences between BS and SJ activations (*p* > 0.05) (Figure 2).

### 3.2. Cross

**Figure 3:**
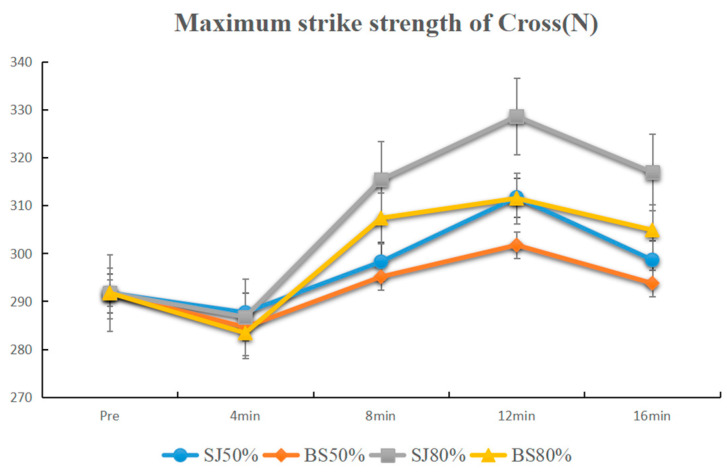
Line plot of the maximum strike strength of cross.

The activation results at 50% 1 RM demonstrated significant differences between 12 min and baseline after BS activation (*p* < 0.01), as well as for SJ activation at both 8 min and 12 min compared to baseline (*p* < 0.01). Adjacent time point comparisons revealed significant differences following BS activation between 4 min and 8 min (4 min < 8 min) and between 12 min and 16 min (12 min > 16 min) (*p* < 0.05), and following SJ activation between 4 min and 8 min (4 min < 8 min), 8 min and 12 min (8 min < 12 min), and 12 min and 16 min (12 min > 16 min) (*p* < 0.05) (Figure 3). Comparisons at the same time points indicated that there were no significant differences between BS and SJ activations (*p* > 0.05).

For the jab, under 50% 1 RM, BS increased maximum-striking strength at 8 and 12 min (*p* < 0.01), while SJ improved at 4, 8, and 12 min (*p* < 0.01), peaking at 8–12 min before declining at 16 min. Under 80% 1 RM, BS improved at 12 min (*p* < 0.01), and SJ at 8 (*p* < 0.05) and 12 min (*p* < 0.01), with high-load activation most effective at 12 min. For the cross, under 50% 1 RM, BS improved at 12 min (*p* < 0.01), and SJ at 8 and 12 min (*p* < 0.01). Under 80% 1 RM, both BS and SJ increased maximum-striking strength at 8, 12, and 16 min (*p* < 0.01), with BS less effective than SJ at 12 min (*p* < 0.05). Adjacent time points showed significant differences between 8 and 12 and 12–16 min (Table 1).

### 3.3. Speed Strike Strength

#### 3.3.1. Jab

The activation results at 50% 1 RM showed no significant differences between the baseline and all time points for both BS and SJ activations (*p* > 0.05). Comparative analysis of adjacent time points revealed a significant difference between 12 min and 16 min after SJ activation (*p* < 0.01, 12 min > 16 min). Comparative analysis at the same time points indicated a significant difference between BS and SJ activations at 12 min with BS being less than SJ (*p* < 0.01).

**Figure 4:**
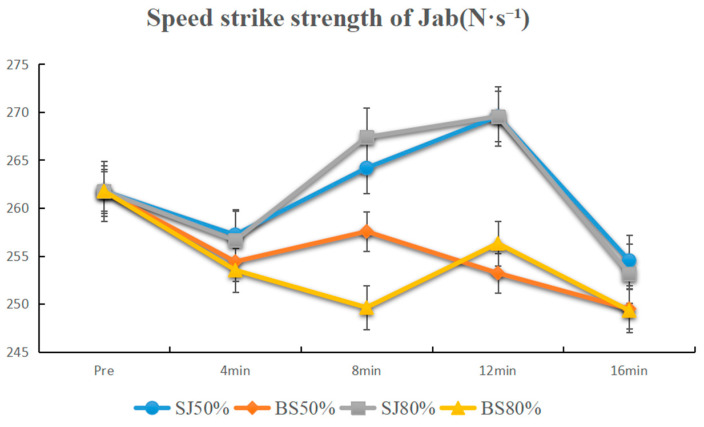
Line plot of the speed strike strength of jab.

The activation results at 80% 1 RM indicated a significant difference between 4 min and the baseline after BS activation (*p* < 0.05), and a significant difference between 12 min and baseline for SJ activation (*p* < 0.01). Comparative analysis of adjacent time points revealed significant differences after SJ activation between 4 min and 8 min (4 min < 8 min) and between 12 min and 16 min (12 min > 16 min) (*p* < 0.01). Comparative analysis at the same time points indicated a significant difference between BS and SJ activations at 8 min, with BS being less than SJ (*p* < 0.01) (Figure 4).

#### 3.3.2. Cross

The activation results at 50% 1 RM showed no significant differences between the baseline and all time points for both BS and SJ activations (*p* > 0.05). Comparative results of adjacent time points indicated that there were no significant differences between each pair of adjacent time points (*p* > 0.05). Comparative analysis at the same time points revealed no significant differences between BS and SJ (*p* > 0.05).

**Figure 5:**
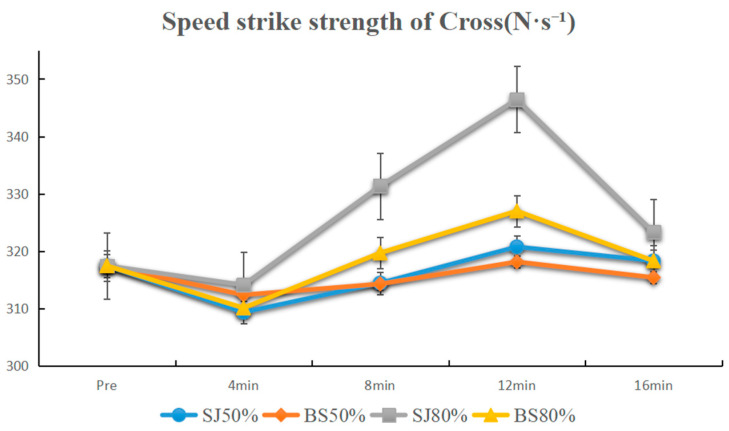
Line plot of the speed strike strength of cross.

The activation results at 80% 1 RM showed no significant differences between the baseline and all time points after BS activations (*p* > 0.05), and a significant difference between 8 min, 12 min and baseline for SJ activation (*p* < 0.01). The comparative results of adjacent time points indicated that significant differences existed between each pair of adjacent time points (*p* < 0.01, 4 min < 8 min < 12 min < 16 min). Comparative analysis at the same time points revealed a significant difference between BS and SJ at 12 min time point, with BS being less than SJ (*p* < 0.01) (Figure 5).

For the jab, under 50% 1 RM, neither BS nor SJ improved speed-striking strength compared to baseline (*p* > 0.05), but SJ showed significant difference between 12 and 16 min (*p* < 0.01), with BS less effective than SJ at 12 min (*p* < 0.01). Under 80% 1 RM, BS improved speed-striking strength at 4 min (*p* < 0.05), while SJ improved at 12 min (*p* < 0.01). SJ also showed significant differences between 4–8 and 12–16 min (*p* < 0.01), with BS less effective than SJ at 8 min (*p* < 0.01). For the cross, under 50% 1 RM, neither BS nor SJ showed significant improvements (*p* > 0.05). Under 80% 1 RM, SJ improved speed-striking strength at 8 and 12 min (*p* < 0.01), with significant differences between 4–8 and 12–16 min (*p* < 0.01), and BS less effective than SJ at 12 min (*p* < 0.01) (Table 2).

## 4. Discussion

The purpose of this study was to compare the effects of different intensities and movement patterns on the transfer of strength in male boxers. The results indicate that using BS_50%_, BS_80%_, SJ_50%_, and SJ_80%_ all resulted in maximum enhancement of the maximum-striking force for jab and cross in boxers within 12 min post-induction. BS_50%_, BS_80%_, and SJ_50%_ did not effectively enhance the rapid striking force of the boxers. However, SJ_80%_ led to the most significant increase in the rapid force of both jab and cross within 12 min post-induction. Notably, significant enhancement was demonstrated earlier in the cross (8 min) than in the jab (12 min).

Previous studies have reported that both heavy-resistance exercises (such as back squat, BS) and ballistic exercises (such as squat jump, SJ) are effective for inducing PAPE, but the time course and magnitude of the effect differ across modalities. For instance, BS-induced PAPE has often been observed at longer recovery intervals (8–12 min), particularly in trained athletes, due to greater neuromuscular fatigue accompanying the higher loads [12,20]. In contrast, SJ tends to elicit PAPE at shorter recovery times (3–7 min), as its ballistic nature produces lower fatigue while still enhancing neural activation and motor unit recruitment [13,21]. The present findings, showing significant improvements primarily after 8–12 min, align more closely with BS-based protocols, suggesting that load intensity and exercise modality critically influence the temporal profile of PAPE. Differences between studies may also stem from sport-specific demands and athlete characteristics; for example, elite combat-sport athletes often demonstrate greater resistance to fatigue and faster recovery, which could shift the optimal window of PAPE compared to athletes in team sports.

### 4.1. Maximum Strike Strength

The action of a straight punch in boxing involves lower limb push, torso rotation, and arm extension, constituting a complex movement process [22]. In boxing, the straight punch is an open chain movement. In open chain movements, the lower limb chain is initiated first, transferring momentum to the upper limb chain through the torso chain. This results in a sequential acceleration from proximal to distal segments [23,24]. Therefore, the lower limbs are the primary source of striking power for boxers, and lower limb explosive power directly affects the effectiveness of the strike [25,26]. Studies have shown that the striking force of the upper limb is closely related to lower limb explosive power, exhibiting a significant positive correlation [27,28]. Additionally, Giovani et al. [29] and Lenetsky [30] have confirmed from various perspectives that the higher the lower limb strength level of male boxers, the greater their striking force. Filimonov et al. pointed out that the contribution of lower limb strength to striking force increases with the level of athletes, and elite athletes’ contribution rate reaches 38.6%, which is higher than that of the torso and arms [25].

This study utilized BS and SJ as inducing methods. According to Loturco et al. [31], BS and SJ can effectively enhance the lower limb explosive power, consequently improving the upper limb striking force. Furthermore, the driving force of the lower limbs during the punching action mainly comes from the peak ground reaction force. SJ and BS exercises can help improve athletes’ knee extension and hip extension strength, leading to an enhancement in peak ground reaction force [30,31]. Furthermore, the muscle activation threshold will decrease as the speed of muscle contraction accelerates. Among them, SJ has a fast contraction speed and can effectively recruit type II muscle fibers. On the other hand, BS, despite having a slower contraction speed and a deceleration phase during the movement, can also activate type II muscle fibers to a certain extent, in accordance with the “size principle” of skeletal muscle fiber recruitment [7,32]. It can be seen that both SJ and BS can effectively activate type II muscle fibers. In this study, both BS and SJ can increase the maximum-striking force of the athletes’ front and rear hand straight punches. The reason for this improvement is that both BS and SJ can enhance lower limb explosive power and activate type II muscle fibers.

This study’s significant improvement in maximum-striking force and the timing of the peak enhancement are consistent with previous research [33], and align with the trend of increased lower limb explosive power. A study has indicated [34] that when the recovery interval is between 8 and 12 min, the enhancement of jumping ability exhibits a moderate effect size (0.24). These findings corroborate the results of Kilduff et al. [35], which clearly illustrate that PAPE surpasses fatigue, indicating a potential time window for enhancing athletic performance. Due to the previously mentioned contraction-induced myosin light chain phosphorylation, some of the calcium released from the sarcoplasmic reticulum during the active phase will bind with calmodulin. This binding stimulates a higher sensitivity of the cross-bridges to calcium, thereby enhancing the sarcomere’s ability to generate force in a shorter period of time [36]. Other aspects related to neural mechanisms are hypothesized to include increased recruitment and synchronization of motor units, as well as a reduction in presynaptic inhibition [37]. However, these mechanisms still require further experimental validation.

### 4.2. Speed Strike Strength

Straight punches are the most commonly used techniques in boxing competitions [38]. After the adoption of the ten-point scoring system in 2013, which replaced the previous “point scoring” method with a more comprehensive approach to determine the winner, the significance of straight punches in boxing matches was further enhanced. Effective punches are a crucial criterion for assessing overall advantage, and the effectiveness of striking is the primary factor in evaluating the impact of punches. Research has revealed that a boxer’s straight punch action must be completed within 50 to 300 ms, with the highest striking speed reaching 8.16 to 8.9 m/s, striking force up to 3500 to 4800 N, and an average round punch count of 58 to 95 times. This indicates that athletes must not only exert maximum-striking force in a short period but also perform rapid consecutive punches, highlighting the crucial role of rapid strength in the sport of boxing [26,39]. Komi [26] refers to the ability of the neuromuscular system to generate maximum strength within a specified time, also known as power-speed. Power-speed performance comprises starting force, rate of force development, and maximum strength. Therefore, it also depends on muscle cross-sectional area, fiber type composition, and the firing rate of motor neurons [40]. Previous studies have shown a positive correlation between maximum strength and rapid strength. A high level of maximum muscle strength is a prerequisite for high-level rapid strength [41].

In terms of load intensity, a load of 50% 1 RM does not effectively induce PAPE, while SJ_80%_ does. Muscle force output can be regulated by changing the activation frequency of individual motor units or by altering the number of activated motor units. Compared to low-load exercises, high-load resistance training is more likely to cause a rapid increase in strength. The stimulus from high-load resistance training can enhance the firing rate of athletes’ neurons, thereby improving neural recruitment ability [42]. The rapid striking force of boxers is influenced by the firing rate of motor neurons. When the firing rate of neurons increases, their ability to recruit type II muscle fibers is greatly enhanced. Type II muscle fibers have been proven to have greater PAPE potential [43,44,45]. This explains why heavy loads can induce a more intense PAPE, rapidly enhancing the striking force of boxers. In addition, an interesting phenomenon was also found: the significant enhancement time of the cross (8 min) occurs earlier than that of the jab (12 min). This may be due to the fact that, compared to the jab, the cross involves more torso rotation and greater contribution from leg strength during the punching process. Moreover, in this study, all the athletes are national-level athletes, demonstrating higher leg power and improved coordination between body parts [25]. The earlier enhancement of the cross may thus be explained.

In terms of movement patterns, we found that at the load intensity of 80% 1 RM, only SJ had a significant impact on the rapid striking force of boxers, while BS_80%_ did not. BE can be defined as the intention to move with maximum velocity [46]. BE must include the action of the body leaving the ground during a jump or the projectile leaving the hand during a throw [7]. BE eliminates the braking phase associated with HRE, which can increase the relative duration of positive acceleration, thereby promoting greater force output and muscle activation [47]. SJ utilized in this study is a prevalent form of BE. The explosive nature of SJ can swiftly activate the neuromuscular system, leading to increased power output [7,48]. Therefore, the study explains that BS_80%_ did not effectively enhance the rapid striking force of boxers, while SJ_80%_ did.

### 4.3. Limitations

A possible explanation for the observed PAPE effects lies in neuromuscular mechanisms, such as enhanced motor unit synchronization and reduced presynaptic inhibition, which may facilitate greater force output. However, it should be noted that the present study did not include direct neurophysiological measurements (e.g., electromyography or H-reflex assessments) to confirm these mechanisms. Therefore, our interpretation remains speculative, and future studies incorporating EMG or other neural monitoring techniques are needed to verify the underlying mechanisms.

## 5. Conclusions

The results of this study demonstrate that various activation methods have different effects on the maximum-striking force and rapid striking force of male boxers’ front and rear straight punches at different activation intensities and time points. The impact of PAPE on the punching force of boxer’s peaks at 12 min, with this peak being particularly influenced by the intensity and method of activation. The activation effect of higher loads is superior to that of medium and low loads. This is evident in a shorter time frame for significant improvement in striking ability and a longer-lasting effect. High-load SJ provides a significant advantage in enhancing the rapid striking force of boxers in the short term.

## Figures and Tables

**Table 1:** The maximum strike strength of jab and cross (N).

Condition	Pre	4 min	8 min	12 min	16 min
Jab SJ50%	237.79 ± 3.79 (0.70)	231.83 ± 4.40 ** (0.82)	243.83 ± 4.18 **^&&^ (0.78)	246.31 ± 3.77 ** (0.70)	235.62 ± 4.36 ^&&^ (0.81)
Jab BS50%		235.83 ± 4.17 (0.77)	241.72 ± 3.61 **^&&^ (0.67)	244.69 ± 4.44 ** (0.82)	239.28 ± 4.64 ^&^ (0.86)
Jab SJ80%		241.45 ± 4.07 (0.76)	243.66 ± 4.01 * (0.74)	248.93 ± 4.01 **^&^ (0.74)	239.69 ± 4.46 ^&&^ (0.83)
Jab BS80%		238.72 ± 3.95 (0.73)	242.14 ± 4.94 ^&^ (0.92)	245.41 ± 4.13 ** (0.77)	237.21 ± 4.86 ^&&^ (0.90)
Cross SJ50%	291.72 ± 6.45 (1.20)	287.66 ± 5.92 (1.10)	298.24 ± 6.42 **^&^ (1.19)	311.66 ± 5.98 **^&^ (1.11)	298.62 ± 6.21 ^&^ (1.15)
Cross BS50%		284.59 ± 6.36 (1.18)	295.10 ± 7.00 ^&^ (1.30)	301.72 ± 7.15 ** (1.33)	293.76 ± 6.57 ^&^ (1.22)
Cross SJ80%		286.69 ± 6.94 (1.29)	315.35 ± 6.63 **^&&^ (1.23)	328.59 ± 5.99 **^&&#^ (1.11)	316.90 ± 7.63 **^&&^ (1.42)
Cross BS80%		283.38 ± 6.54 (1.21)	307.35 ± 6.80 **^&&^ (1.26)	311.48 ± 6.05 ** (1.12)	304.86 ± 6.23 ** (1.16)

Notes: * Indicates a statistically significant difference compared to the baseline (pre) (* for *p* < 0.05, ** for *p* < 0.01); ^&^ indicates a statistically significant difference compared to the previous time point (^&^ for *p* < 0.05, ^&&^ for *p* < 0.01); ^#^ indicates a statistically significant difference between BS and SJ at the same 1 RM (^#^ for *p* < 0.05). Values are expressed as Mean ± SD (with SEM in parentheses).

**Table 2:** The speed strike strength of jab and cross (N·s^−1^).

Condition	Pre	4 min	8 min	12 min	16 min
Jab SJ50%	261.76 ± 6.39 (1.19)	257.21 ± 4.70 (0.87)	264.17 ± 4.37 (0.81)	269.55 ± 3.92 ^##^ (0.73)	254.52 ± 4.59 ^&&^ (0.85)
Jab BS50%		254.41 ± 4.43 (0.82)	257.55 ± 3.80 (0.71)	253.21 ± 4.76 (0.88)	249.48 ± 4.90 (0.91)
Jab SJ80%		256.62 ± 4.26 (0.79)	267.38 ± 4.23 ^&&##^ (0.79)	269.55 ± 4.27 ** (0.79)	253.17 ± 4.51 ^&&^ (0.84)
Jab BS80%		253.52 ± 4.15 * (0.77)	249.62 ± 5.23 (0.97)	256.31 ± 4.10 (0.76)	249.31 ± 5.19 (0.96)
Cross SJ50%	317.45 ± 9.26 (1.72)	309.35 ± 6.17 (1.15)	314.45 ± 6.69 (1.24)	320.76 ± 6.16 (1.14)	318.31 ± 6.37 (1.18)
Cross BS50%		312.38 ± 6.65 (1.23)	314.28 ± 7.38 (1.37)	318.14 ± 7.51 (1.39)	315.41 ± 6.76 (1.26)
Cross SJ80%		314.10 ± 7.35 (1.36)	331.35 ± 7.02 **^&&^ (1.30)	346.45 ± 6.15 **^&#^ (1.14)	323.31 ± 7.71 ^&&^ (1.45)
Cross BS80%		310.10 ± 6.83 (1.27)	319.69 ± 7.13 (1.32)	327.00 ± 6.36 (1.18)	318.31 ± 6.28 (1.17)

Notes: * Indicates a statistically significant difference compared to the baseline (pre) (* for *p* < 0.05, ** for *p* < 0.01); ^&^ indicates a statistically significant difference compared to the previous time point (^&^ for *p* < 0.05, ^&&^ for *p* < 0.01); ^#^ indicates a statistically significant difference between BS and SJ at the same 1 RM (^#^ for *p* < 0.05, ^##^ for *p* < 0.01). Values are expressed as Mean ± SD (with SEM in parentheses).

## Data Availability

Further inquiries can be directed to the corresponding author.
